# The putative Na^+^/Cl^−^-dependent neurotransmitter/osmolyte transporter inebriated in the *Drosophila* hindgut is essential for the maintenance of systemic water homeostasis

**DOI:** 10.1038/srep07993

**Published:** 2015-01-23

**Authors:** Zhuo Luan, Caitlin Quigley, Hong-Sheng Li

**Affiliations:** 1 Department of Neurobiology, University of Massachusetts Medical School, Worcester, MA 01605, USA

## Abstract

Most organisms are able to maintain systemic water homeostasis over a wide range of external or dietary osmolarities. The excretory system, composed of the kidneys in mammals and the Malpighian tubules and hindgut in insects, can increase water conservation and absorption to maintain systemic water homeostasis, which enables organisms to tolerate external hypertonicity or desiccation. However, the mechanisms underlying the maintenance of systemic water homeostasis by the excretory system have not been fully characterized. In the present study, we found that the putative Na^+^/Cl^−^-dependent neurotransmitter/osmolyte transporter inebriated (ine) is expressed in the basolateral membrane of anterior hindgut epithelial cells. This was confirmed by comparison with a known basolateral localized protein, the α subunit of Na^+^-K^+^ ATPase (ATPα). Under external hypertonicity, loss of ine in the hindgut epithelium results in severe dehydration without damage to the hindgut epithelial cells, implicating a physiological failure of water conservation/absorption. We also found that hindgut expression of ine is required for water conservation under desiccating conditions. Importantly, specific expression of ine in the hindgut epithelium can completely restore disrupted systemic water homeostasis in *ine* mutants under both conditions. Therefore, ine in the *Drosophila* hindgut is essential for the maintenance of systemic water homeostasis.

Water homeostasis is essential for the survival of all organisms. The mammalian kidney and the Malpighian tubule and hindgut of insects play indispensable roles in maintaining water homeostasis over a wide range of external or dietary osmolarities. These organs can increase water conservation and absorption to maintain systemic water homeostasis, which enables organisms to tolerate external hypertonicity or desiccation^1^. The mammalian kidney regulates water balance mainly through the antidiuretic hormone (ADH)^2^,^3^,^4^,^5^, which enhances water absorption. Failure of antidiuretic mechanisms can result in disrupted systemic water homeostasis, causing pathological conditions like Diabetes Insipidus^6^. Although antidiuretic factors for the enhancement of water absorption, such as Schgr-ITP and CAPA-related peptides, are also present in insects^7^,^8^,^9^,^10^,^11^,^12^,^13^,^14^,^15^,^16^,^17^, the mechanisms of water conservation and absorption in the excretory system are not fully characterized, especially in *Drosophila*.

Previous studies have shown that loss of the putative Na^+^/Cl^−^-dependent neurotransmitter/osmolyte transporter inebriated (ine) causes hypersensitivity to dietary hypertonicity in *Drosophila*; however, the mechanism underlying this effect remains unknown^18^. Ine is a member of the Na^+^/Cl^−^-dependent neurotransmitter/osmolyte transporter family, which is conserved across invertebrates and vertebrates^18^,^19^. Members of this family share several common structural features, including 12 transmembrane domains flanked by intracellular N and C termini, and an extracellular loop between the third and fourth transmembrane domains^20^. These proteins play critical roles in neurotransmission, as well as cellular and systemic homeostasis, by transporting neurotransmitters, osmolytes, and energy metabolites across the plasma membrane. There is sequence similarity between ine and the betaine/GABA transporter (BGT1), a mammalian member of the Na^+^/Cl^−^-dependent neurotransmitter/osmolyte transporter family. Both BGT1 and ine are expressed in the central nervous system (CNS), as well as organs that perform water absorption, and both are involved in the control of neuronal excitability and tolerance to hypertonicity^18^,^19^,^21^,^22^,^23^. This suggests that these two proteins may function through a similar mechanism. Betaine, an active organic compound, is the substrate of BGT1 in renal medullary cells; however, the substrate of ine has yet to be identified. Betaine, like other intracellular organic osmolytes, can protect cells from external hypertonicity by balancing high extracellular osmolarity and preserving cell volume without interfering with cell function^24^,^25^. However, no direct genetic evidence supports the osmoprotective function of the BGT1-mediated accumulation of betaine in renal medullary cells^26^,^27^. Specifically, BGT1 knockout mice are healthy, and renal medullary cells appear to be normal in the hypertonic environment of the renal medulla^26^. Therefore, the physiological function of the Na^+^/Cl^−^-dependent neurotransmitter/osmolyte transporter in the excretory system remains to be elucidated.

By investigating the function of ine in *Drosophila*, an excellent genetic model in which gene expression can be evaluated and manipulated *in vivo*, we may begin to understand the physiological function of Na^+^/Cl^−^-dependent neurotransmitter/osmolyte transporters, including BGT1, in the excretory system. In this study, we elucidate the role of ine in the *Drosophila* hindgut, and reveal a novel mechanism mediated by ine for the maintenance of systemic water homeostasis.

## Results

### Ine is expressed in the basolateral membrane of adult hindgut epithelial cells and co-localizes with Na^+^-K^+^ ATPase

**Figure 1:**
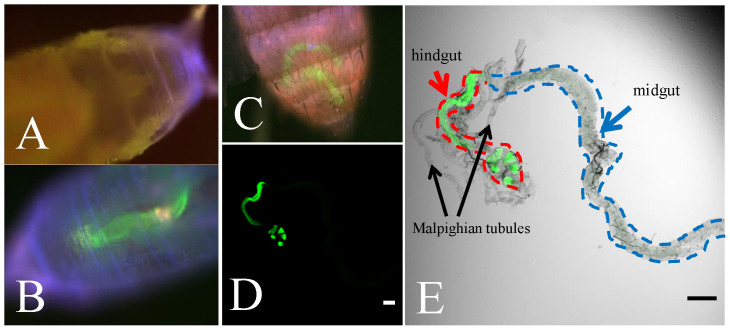
The hindgut-specific expression pattern of the *hindgut-Gal4* line as visualized using UAS-GFP. In pupae, GFP signal is detected in the posterior part (B) but not in the anterior part (A). The GFP signal is detected in the abdomen of the adult fly (C). The *hindgut-Gal4* is specifically expressed in the hindgut but not in the midgut and Malpighian tubules (D and E). Scale bars: 100 μm.

**Figure 2:**
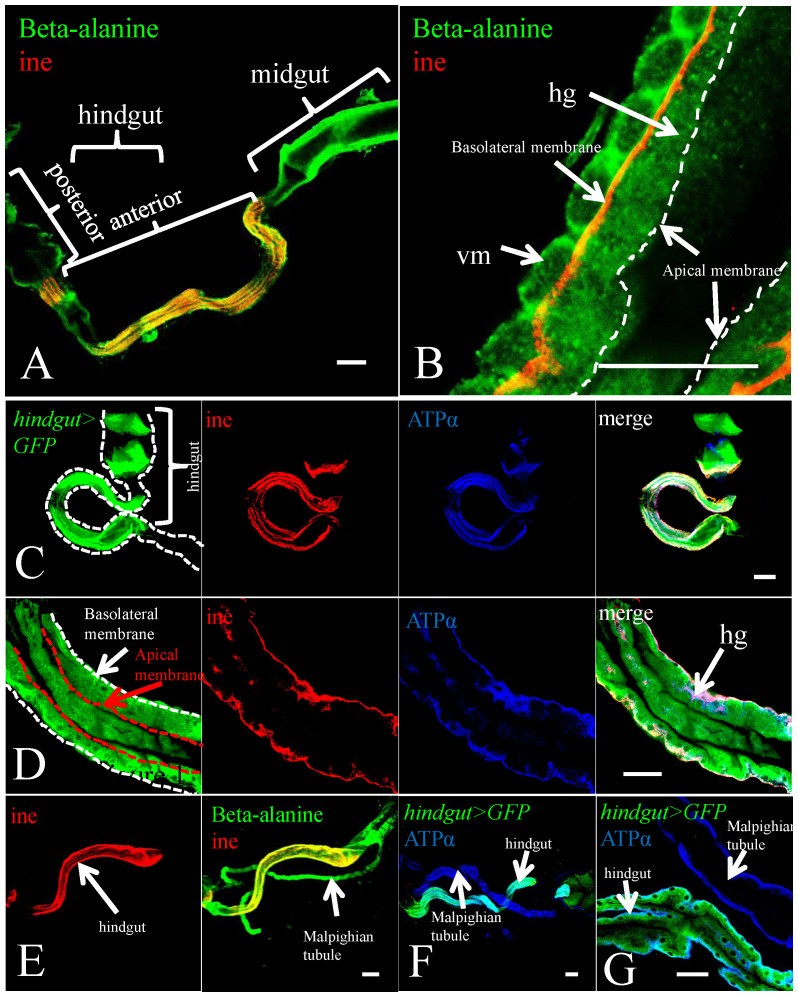
Ine is localized at the basolateral membrane of the hindgut epithelial cells. The hindguts were stained with an anti-ine antibody (red). (A) and (B), both the hindgut epithelium (hg) and visceral muscle layer (vm) were labeled with a β-alanine antibody (green). Ine localizes to the basolateral membrane, but not the apical membrane, of the anterior hindgut epithelial cells. (C) and (D), the hindguts of *hindgut-Gal4* *>* *UAS-GFP* flies were stained with an anti-ine antibody (red) and an antibody against the α subunit of Na^+^-K^+^ ATPase (ATPα, blue). ATPα signal co-localizes with ine in the hindgut epithelial cells. (E), Ine is not expressed in the Malpighian tubules. (F) and (G), ATPα is localized to the basolateral membrane of the hindgut and Malpighian tubules. Scale bars: a, c, e and f, 100 μm; b, d and g, 50 μm.

Although ine mRNA is observed in the hindgut and Malpighian tubules of *Drosophila* embryos via whole-mount *in situ* hybridization^18^,^19^, the expression pattern of ine protein in the adult fly is still uncharacterized. To answer this question, we generated an anti-ine antibody to observe the subcellular localization of ine, and *hindgut-Gal4* to label hindgut epithelial cells (Fig. 1). The hindgut is divided into two sections: anterior (the ileum) and posterior (the rectum). We performed double-immunofluorescent staining on the gut and Malpighian tubules using antibodies against β-alanine, which generally labels the structure of the gut, and ine. We found that ine is specifically expressed in the basolateral membrane of the anterior hindgut epithelium, but not in other parts of the hindgut or in the Malpighian tubules (Fig. 2A, B and E)^28^. This expression pattern conflicts with previous reports of ine mRNA distribution^29^; however, the discrepancies may be due to various biological factors such as complex gene regulatory mechanisms^30^.

The subcellular localization of ine was further confirmed by comparison with the α subunit of Na^+^-K^+^ ATPase (ATPα), which is known to localize to the basolateral membrane in Malpighian tubules^31^. We observed that ATPα is also localized to the basolateral membrane of the hindgut epithelium using an anti-ATPα antibody (Fig. 2G). We labeled all membranes of hindgut epithelial cells by driving membrane-bound GFP with *hindgut-Gal4*, and the basolateral membrane with anti-ATPα antibody. Upon co-staining with anti-ine antibody, we found that ine completely co-localized with ATPα in the basolateral membrane of the hindgut epithelium (Fig. 2C and D). BGT1 also localizes to the basolateral membrane of renal medullary cells, which allows the cells take up betaine from circulation rather than the medullary lumen^26^. Similarly, ine might transport an as yet unknown osmolyte into hindgut epithelial cells from the hemolymph, rather than the hindgut lumen.

### Ine in the hindgut epithelium is essential for tolerance of dietary hypertonicity in *Drosophila*

**Figure 3:**
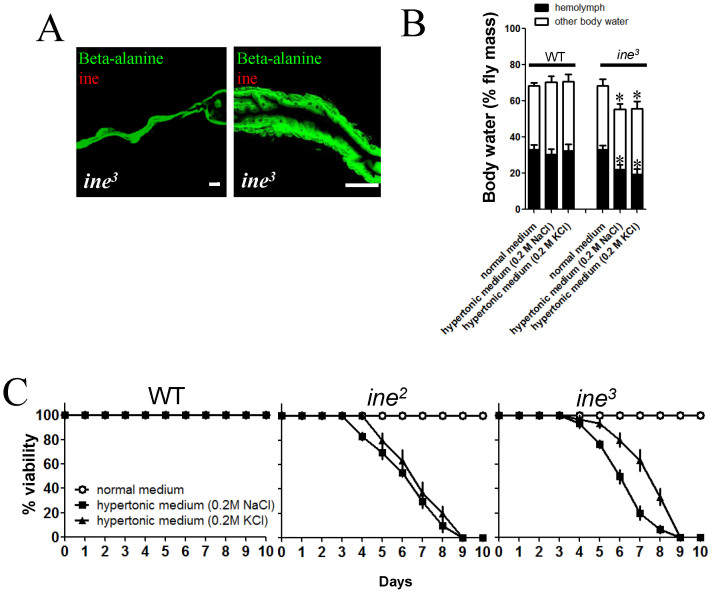
The loss of ine in the hindgut epithelium disrupts systemic water homeostasis, which leads to death under conditions of external hypertonicity. (A), the ine signal is completely abolished in the hindgut epithelium of the null mutant *ine*^*3*^. Scale bars: 100 μm. (B), when maintained on normal medium, *ine*^*3*^ mutants have a similar hemolymph volume and total body water content to the WT flies. However, the hemolymph volume and total body water content of *ine*^*3*^ mutants declined dramatically, while those of the WT flies were not affected, when the flies were maintained on hypertonic media (n = 5, *t-*tests, two tails). Each bar represents the mean ± S.E.M. Asterisk (*): *p* < 0.01. (C), *ine* mutant flies exhibited no lethality when maintained for up to 10 days on normal medium, while those maintained on hypertonic media died within 10 days. Dietary hypertonicity does not affect WT flies. Each point represents the mean ± S.E.M. The data are representative of three independent experiments.

**Figure 4:**
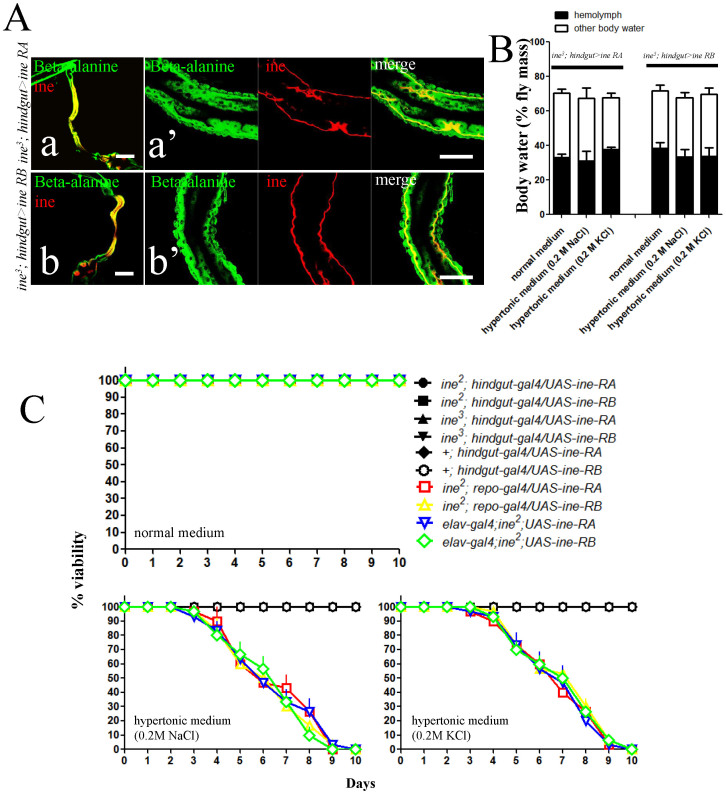
Specific overexpression of ine in the hindgut epithelium can restore disrupted systemic water homeostasis and rescue lethality under dietary hypertonicity in *ine* mutants. (A), overexpression of the RA or RB isoforms of ine using *hindgut-Gal4* results in targeting to the basolateral membrane of the hindgut epithelial cells in the *ine*^*3*^ mutant. Scale bars: a and b, 200 μm; a′ and b′, 100 μm. (B), overexpression of the RA or RB isoforms of ine in the hindgut epithelium can completely restore the hemolymph volume and total body water content of *ine*^*3*^ flies to normal levels (n = 5, *t-*tests, two tails). Each bar represents the mean ± S.E.M. (C), overexpression of the RA or RB subunit of ine exclusively in hindgut epithelial cells completely rescued the lethality of *ine*^*2*^* or ine*^*3*^ mutants maintained on hypertonic media. Each point represents the mean ± S.E.M. The data are representative of three independent experiments.

Previous studies have shown that loss of ine causes hypersensitivity to dietary hypertonicity in *Drosophila*. We sought to repeat these findings. To characterize the differential tolerance of dietary hypertonicity between WT flies and *ine* mutants, we prepared fly food media with a 0.2 M salt solution in place of water. Consistent with previous findings^18^, we observed a sensitivity to dietary hypertonicity in *ine* mutants. We studied flies bearing two different mutations in the ine gene, *ine*^*2*^ and *ine*^*3*^, and found in both cases that flies maintained on normal medium exhibited no lethality, whereas those maintained on hypertonic media died within 10 days. In contrast, dietary hypertonicity had no effect on the viability of WT flies (Fig. 3C). Because ine is expressed in the CNS as well as the hindgut, we tested whether the intolerance to dietary hypertonicity was due to the loss of ine specifically in the CNS or the hindgut tissue. Ine has 2 isoforms, RA and RB, which may have different functions. We rescued the *ine*^*2*^ and *ine*^*3*^ mutant phenotypes by overexpressing either the RA or RB isoform using *hindgut-Gal4*. Overexpression of either isoform resulted in localization of the protein to the basolateral membrane (Fig. 4A), similar to the endogenous distribution pattern (Fig. 2). This result suggests that the overexpressed protein functions normally. Both the RA and RB isoform were sufficient to rescue lethality in *ine*^*2*^ and *ine*^*3*^ flies maintained on hypertonic media. However, expression of either the RA or RB isoform in neurons or glia using *elav-* and *repo-Gal4*, respectively, did not rescue lethality in mutants fed on hypertonic media (Fig. 4C). These results indicate that ine is required in the hindgut epithelium, but not the CNS, for tolerance to dietary hypertonicity.

### Ine is not involved in the osmoprotective response to external hypertonicity in anterior hindgut epithelial cells

**Figure 5:**
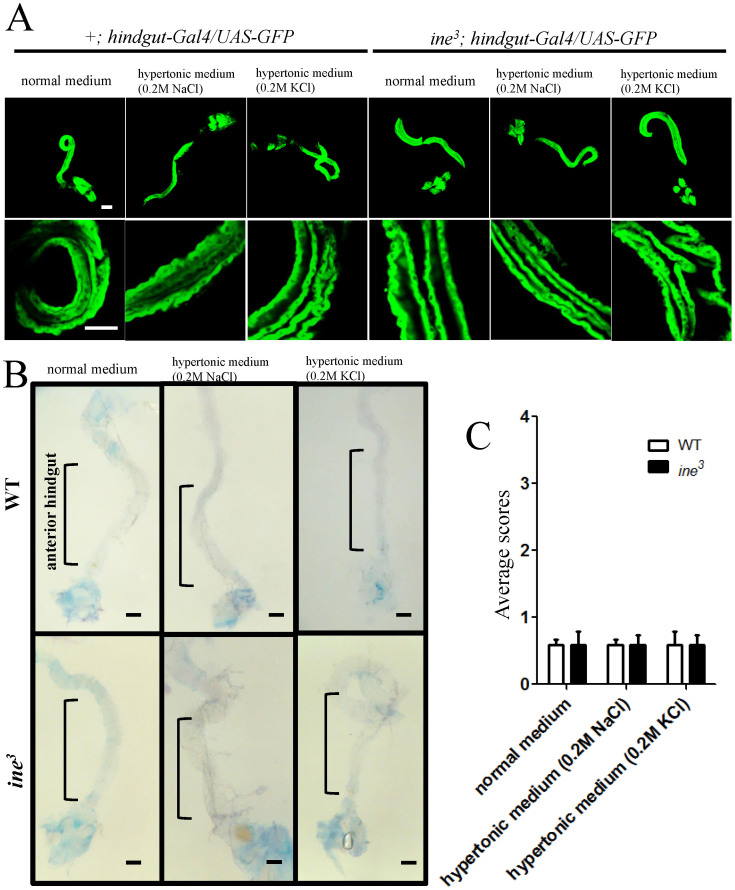
External hypertonicity does not damage anterior hindgut epithelial cells in the absence of ine. (A), hindgut epithelial cells were labeled with GFP in WT or *ine*^*3*^ backgrounds using *hindgut-Gal4*. Maintenance on hypertonic media for 4 days did not affect the expression of GFP in the hindgut epithelial cells of WT or *ine*^*3*^ flies, which indicates that the cells are intact. (B), hindgut tissue of WT and *ine*^*3*^ flies maintained on normal or hypertonic media for 4 days presented minimal Trypan blue staining. (C), quantification of Trypan blue staining in the anterior hindgut. No significant differences were observed in the viability of the anterior hindgut between WT and *ine*^*3*^ flies maintained on normal or hypertonic media (n = 5, *t-*tests, two tails). Each bar represents the mean ± S.E.M. Scale bars: 100 μm.

Huang et al. postulated that elevated intracellular levels of Na^+^ and K^+^ in hindgut epithelial cells in response to external hypertonicity would be lethal, either through a necrotic or apoptotic mechanism, unless normal intracellular Na^+^ and K^+^ levels could be restored. They proposed an osmoprotective role for ine, in which an osmolyte transported by ine increased intracellular molality thus allowing Na^+^ and K^+^ to move out of the cell, and returning cell volume and ion concentration to normal physiological levels^18^. If this osmoprotective theory is correct, hindgut epithelial cells without ine would undergo necrotic or apoptotic cell death under conditions of external hypertonicity. Therefore, we examined whether anterior hindgut epithelial cells were damaged by external hypertonicity in the absence of ine. We labeled hindgut epithelial cells with GFP using *hindgut-Gal4* in a WT or *ine*^*3*^ background and maintained the flies on normal or hypertonic media. After 4 days, we dissected out the hindgut and were able to detect GFP signal in the hindgut epithelium. This result demonstrates that external hypertonicity does not affect GFP expression in the hindgut epithelial cells of WT or mutant flies (Fig. 5A), and indicates that epithelial cells in those flies were healthy. To further examine tissue damage, including necrotic and apoptotic cell death, we stained the hindgut with Trypan Blue, a dye that is excluded from intact cells but is rapidly absorbed by dead or dying cells^32^. In normal conditions, WT gut showed minimal staining in the ileum and moderate staining in the rectum, which may be due to desiccation damage of remnants of muscle, fat, and connective tissue surrounding it. WT and *ine*^*3*^ adult flies were maintained on normal or hypertonic media for 4 days, after which hindguts were dissected out and stained with Trypan Blue. Both WT and *ine*^*3*^ flies exhibited little or no Trypan Blue staining in the anterior hindgut (Fig. 5B and C), indicating that without ine, anterior hindgut epithelial cells are not damaged by external hypertonicity, and that the osmoprotective response of the epithelial cells against external hypertonicity is normal. Therefore, ine does not function as an osmoprotector in anterior hindgut epithelial cells. We propose that ine has a direct, physiological role in water conservation/absorption that is not secondary to protection of the hindgut epithelium from damage.

### The expression of ine in hindgut epithelium is indispensable for the maintenance of systemic water homeostasis

The hindgut is important for fluid absorption in many insects^33^; however, this function has never been demonstrated in the hindgut of adult *Drosophila*. Considering its specific expression in the hindgut and the hypersensitivity of mutants to dietary hypertonicity, we hypothesized that ine in the hindgut epithelium is essential for water conservation/absorption in response to external hypertonicity. Therefore, we examined the volume of hemolymph and the total body water content in WT, *ine*^*3*^, and mutant flies rescued with either the RA or RB isoform of ine. Adult flies were maintained on normal or hypertonic media for 4 days, after which hemolymph volume and total body water content of individual flies were quantified. When maintained on normal medium, *ine*^*3*^ flies had a similar hemolymph volume and total body water content to WT flies (Fig. 3B). Under external hypertonicity, the hemolymph volume and total body water content of the *ine*^*3*^ flies declined dramatically while those of WT flies were not affected. Overexpression of the RA or RB isoform in the hindgut epithelium by *hindgut-Gal4* completely and independently rescued the severe loss of body water in mutants (Fig. 4B), indicating that the two isoforms have similar functions. These results suggest that ine in the hindgut epithelium may mediate water conservation/absorption, which is essential for the maintenance of systemic water homeostasis under external hypertonicity. In humans, losing approximately 20% of the body's water content is known to cause delirium, coma and death^34^,^35^. Therefore, the severe dehydration caused by the failure of water conservation/absorption may be the primary reason for the death of *ine* mutants under external hypertonicity.

**Figure 6:**
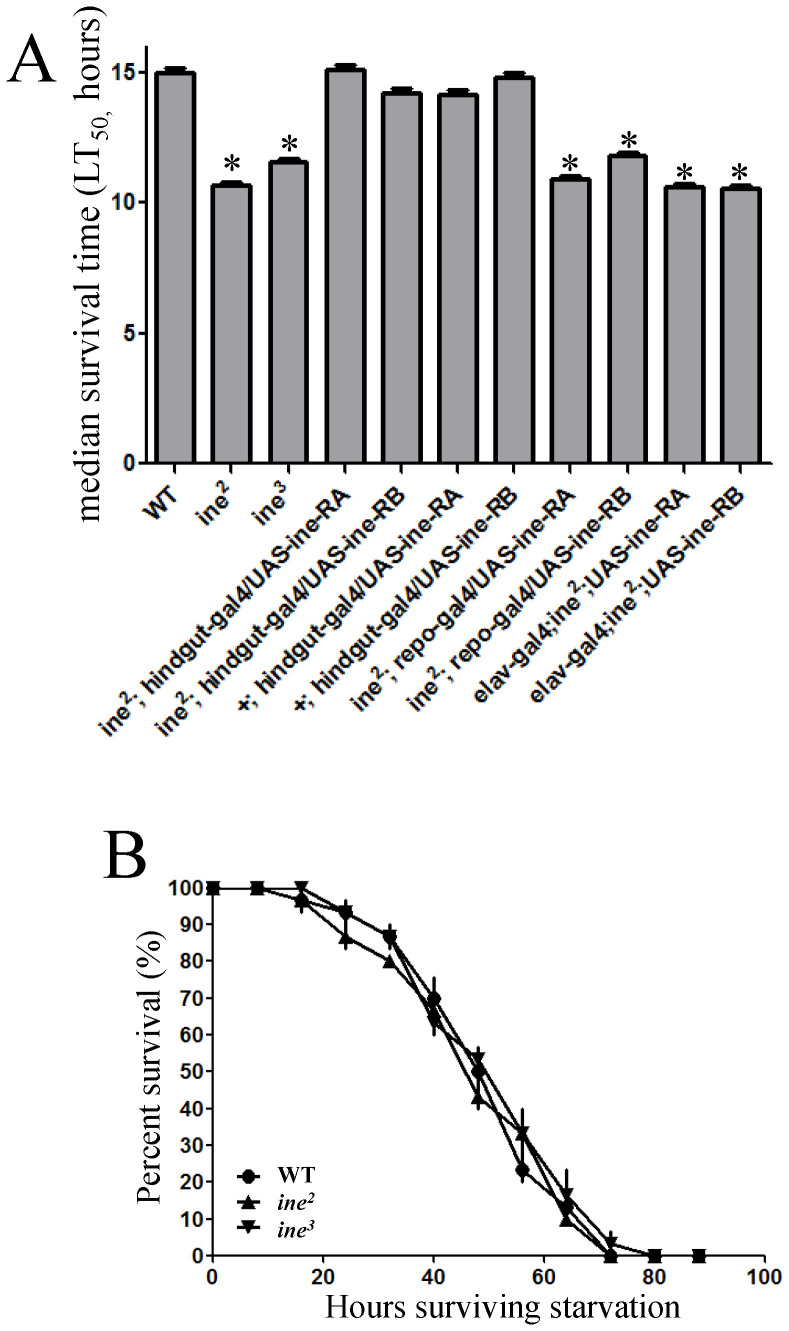
Ine in the hindgut is essential for water conservation under conditions of desiccation. (A), *ine* mutants are more sensitive to desiccation than WT flies. The expression of the RA or RB isoform of ine in the hindgut epithelium by *hindgut-Gal4* but not by *repo-* or *elav-Gal4* can completely rescue the sensitivity of mutants to desiccation. The median survival time (LT_50_) was estimated for different groups. Each bar represents the mean ± S.E.M. Asterisk (*): *p* < 0.01, compared with WT (*t-*tests, two tails). (B), survival curves demonstrate similar starvation resistance between the *ine* mutant and WT flies when supplied with sufficient water. The experiments were performed in triplicate (n = 10 per genotype). The data are the means ± S.E.M.

We questioned whether the mechanism mediated by ine functions under conditions of other than hypertonicity, such as desiccation, in which water is withheld, and starvation, in which flies are only given a water supply. To address this question, we measured the resistance of *ine* mutant flies to desiccation and starvation (Fig. 6). We found that the *ine* mutants were more sensitive than WT flies to desiccation, indicating that the rate of water loss was higher in *ine* mutants than in WT flies. *Drosophila* lose water through three mechanisms: excretion from the mouthparts and anus, cuticular transpiration, and respiratory loss through the spiracles^36^. Water conservation/absorption mediated by ine may reduce the rate of water loss through excretion to combat dehydration. The expression of either the RA or RB isoform of ine in the hindgut epithelium by *hindgut-Gal4*, but not by *repo-* or *elav-Gal4*, completely rescued the sensitivity of mutants to desiccation. In contrast, under hypotonic conditions with only a water supply, mutants and WT flies exhibited a similar resistance to starvation. These results indicate that ine is indispensable for water conservation/absorption under conditions of desiccation but not starvation, and is essential for the maintenance of systemic water homeostasis.

## Discussion

We have demonstrated that the mediation of water conservation/absorption by ine in the hindgut is essential for the maintenance of systemic water homeostasis in *Drosophila*. In insects, systemic water homeostasis is tightly regulated by the excretory system, including the Malpighian tubules and the hindgut, to ensure a constant internal environment^37^. The dynamic balance between Malpighian tubule secretion and hindgut reabsorption, both of which are controlled by diuretic and antidiuretic hormones or factors, maintains water homeostasis in response to fluctuations in external osmotic conditions^7^,^8^,^9^,^10^. However, in adult *Drosophila*, the water conservation/absorption mechanisms of the hindgut have not been elucidated. Our results demonstrate that ine is expressed in the basolateral membrane of the hindgut epithelium, suggesting that ine transports substrate from the hemolymph into hindgut epithelial cells. Surprisingly, under conditions of external hypertonicity, the systemic water homeostasis of *ine* mutant flies is disrupted, whereas that of WT flies is not disturbed. These results demonstrate that hindgut expression of ine mediates water conservation/absorption under external hypertonicity and maintains systemic water homeostasis. These results also suggest possible mechanism for ine function: transport of an osmolyte by ine into the hindgut epithelium increases intracellular molarity, which enhances water conservation/absorption from the hindgut lumen. Such a function would be particularly important in the condition of external hypertonicity, when increased molality in the hindgut lumen prevents osmotic flow of water into hindgut epithelium.

It could be argued that ine functions through an osmoprotective mechanism, in which increased intracellular accumulation of osmolytes mediated by ine protects the hindgut epithelium from cellular death due to extracellular hypertonicity. However, we demonstrate that anterior hindgut epithelial cells are not damaged by external hypertonicity in the absence of ine, suggesting that ine function in water conservation/absorption is not secondary to an osmoprotective effect. We propose the existence of other osmolytes or transporters that function as osmoprotectors, and protect anterior hindgut epithelial cells against lethality under external hypertonicity^26^. The expression of several genes, including some organic transporters, is up-regulated in the hindgut in response to external hypertonicity^38^, supporting this possibility.

Ine protein is expressed solely in the anterior hindgut. The anterior hindgut is an important site of water absorption, as demonstrated in insects other than *Drosophila*. In locusts, isosmotic fluid absorption in the anterior hindgut is driven by an apical membrane electrogenic Cl^−^ pump. The antidiuretic hormone Schgr-ITP acts on the locust hindgut via cyclic AMP and GMP to increase the conductance of both K^+^ and Na^+^ and to stimulate the Cl^−^ pump. As a result of the increased ion uptake, water absorption increases^39^,^40^. It remains unknown, however, whether similar ion-uptake-coupled water absorption mechanisms are present in the *Drosophila* hindgut. We found that loss of ine in the anterior hindgut epithelium causes severe dehydration in response to a hypertonic diet, and higher rates of body water loss under desiccation, which suggests the existence of a new mechanism of water conservation/absorption in the hindgut of *Drosophila* mediated by ine. We propose above that ine transports osmolytes across the plasma membrane from the hemolymph and accumulates osmolytes within the hindgut epithelium, generating an osmotic driving force to conserve/absorb water from hindgut lumen against external hypertonicity. However, this theory lacks an explanation for how water is transferred into the hemolymph from epithelial cells, and to date, the transporter activity of ine has not been confirmed. We cannot rule out the possibility that ine may improve water conservation/absorption through a different, unknown mechanism.

In addition to the anterior hindgut, the Malpighian tubules, rectum, and midgut also contribute to water absorption and conservation in insects under conditions of external hypertonicity or desiccation. During dehydration stress, the modulation of tyramine signaling in *Drosophila* Malpighian tubules enhances conservation of body water^41^. Several anti-diuretic factors acting on the Malpighian tubules have been found. For example, CAPA-1 acts on Ncc69, the Na^+^-K^+^-2Cl^−^ cotransporter, to increase water absorption through an ion uptake coupled mechanism^42^. In addition, PKG, a cGMP-dependent kinase antagonizes the diuretic effects of tyramine and leukokinin^9^. The rectum can also transport water from lumen to the hemolymph^33^,^43^,^44^. In the locust, the chloride transport stimulating hormone (CTSH) acts to increase ion-dependent active transport of fluid from the rectum lumen^45^. Finally, the antidiuretic hormone RhoprCAPA-2 inhibits fluid transport into the midgut lumen in *Rhodnius prolixus* to conserve water^13^. Therefore, ine-mediated water conservation/absorption may not be the only mechanism by which systemic water homeostasis is maintained under external hypertonicity in *Drosophila*.

Water is essential for the proper function of virtually all living cells. Organisms have developed mechanisms in the excretory system to maintain water hemostasis for a constant internal milieu under different external osmotic conditions, such as hypertonicity. Our study reveals that hindgut expression of ine, a putative Na^+^/Cl^−^-dependent neurotransmitter/osmolyte transporter, is indispensable for the maintenance of systemic water homeostasis in *Drosophila*. However, further investigation of the novel mechanism mediated by ine in the hindgut is necessary to fully understand the water conservation and absorption mechanisms of *Drosophila* hindgut, as well as the physiological functions of the members of the Na^+^/Cl^−^-dependent neurotransmitter/osmolyte transporter family.

## Methods

### Fly stocks

Fly stocks were raised on standard cornmeal-agar medium with 12 hr light/12 hr dark cycles at 25°C and 60% humidity. The wild-type (WT) strain used was Canton-Special (Canton-S). The *ine*^*2*^ and *ine*^*3*^ mutants, and the transgenic flies carrying *UAS-ine-RA*, were kindly provided by Dr. Michael Stern^18^. *Repo-Gal4*, *elav-Gal4* and *UAS-GFP* strains were obtained from the *Drosophila* Stock Center in Bloomington. The transgenic flies carrying *UAS-ine-RB* and *hindgut-Gal4* were generated in this study (see below). *hindgut-Gal4* is expressed exclusively in the hindgut epithelial cells of flies as confirmed by *hindgut-Gal4* directed cytoplasmic GFP expression.

### Generation of *hindgut-Gal4* and *UAS-ine-RB* transgenic flies

To generate *hindgut-Gal4*, we selected *Irk2* (CG4370), for which predicted function implied expression in the hindgut. Fragments containing the upstream region (3R: 23518270-23521443) flanking this gene were amplified using PCR from genomic DNA (Primers: forward, 5′-ATGTCAGAGCAATCAACTTCTCTTG-3′; reverse, 5′-TTCTCTAGACGTTTTAACTTCGCGG-3′). The fragments were cloned, sequence-verified, and inserted into the pG4PN vector upstream of the Gal4 cassette. The final DNA construct was injected into *w1118* embryos by BestGene, Inc. (Chino Hills, CA)^46^. We analyzed the expression pattern of *hindgut-Gal4* by driving expression of a UAS-GFP reporter gene, and detecting fluorescence using confocal microscopy of whole mount tissue (Fig. 1). Wild-type ine-RB cDNA was obtained by RT-PCR (Primers: forward, 5′- ATGCCGAACCGCCAGGACTACGAT-3′; reverse, 5′-CTACTGGCCACATGGTCCTCCTGCC-3′), subcloned into a pUAST vector, and injected into *w1118* flies by BestGene, Inc. (Chino Hills, CA) to generate *UAS-ine-RB* transgenic flies.

### Antibodies

The ine antibody was raised in guinea pig against a GST-fused fragment of ine protein (C-terminal portion of ine, 847-943a.a.). The antibody was affinity purified by coupling the antigen to Sepharose 4B. The specificity of the antibody was validated by immunostaining of the null mutant *ine*^*3*^. Rabbit polyclonal Anti-GFP antibody was purchased from Life Technologies. Rabbit polyclonal anti-β alanine antibody (ab37076), purchased from Abcam (Cambridge, MA) was used to label the general gut structure of adult *Drosophila*. Mouse monoclonal antibody α5-IgG, specific for the α-subunit of the Na^+^/K^+^-ATPase, was obtained from The University of Iowa Developmental Studies Hybridoma Bank^47^. All secondary antibodies were purchased from Jackson ImmunoResearch.

### Immunostaining and confocal imaging

Hindgut tissue was prepared for immunostaining as previously described^48^. Briefly, tissue was dissected and fixed in 100 mM glutamic acid, 25 mM KCl, 20 mM MgSO_4_, 4 mM sodium phosphate, 1 mM MgCl_2_, 4% formaldehyde for 30 min. Subsequent rinses, washes and incubations with primary and secondary antibodies were performed in 1X PBS, 0.5% BSA, 0.3% TritonX-100. Tissue was mounted in Vectashield medium (Vector Laboratories). Images were captured using confocal microscopy on an LSM 510 instrument (Zeiss). The following antibodies were used: anti-ine (1:200), anti-GFP (1:500), anti-β Alanine (1:100) and α5 (1:100). Secondary antibodies were used at 1:500 and are as follows: goat anti guinea pig, goat anti-rabbit, and goat anti-mouse IgG conjugated to cy3, Alexa 488 and cy5 respectively.

### Viability assays on hypertonic media

Flies were collected for 4 days following eclosion. Instant fly food medium (Carolina) was prepared according to the manufacturer's instructions. Hypertonic medium was prepared by replacing water with 0.2 M NaCl or KCl solution. Adult flies of the indicated genotype (10 per vial) were maintained on either normal or hypertonic medium for 10 days. Live and dead flies were counted daily. Fly manipulations and assays were conducted at room temperature and ambient humidity^18^.

### Trypan blue staining

WT and *ine*^*3*^ flies were maintained on either normal or hypertonic medium for 4 days. Hindgut tissue was prepared for Trypan blue staining as previously described^32^. Briefly, tissue was dissected in 1X PBS, immersed in 0.2 mg/ml Trypan Blue in 1X PBS, and rotated for 30 min at room temperature. After washing in PBS for 30 min, the tissue was immediately scored for Trypan Blue staining of the anterior hindgut. Scoring was based on an index of the anterior hindgut: no color, 0; any blue, 1; darkly stained nuclei, 2; large patches of darkly stained cells, 3; or complete staining of most cells in the tissue, 4.

### Hemolymph volume and body water measurement

Hemolymph volume and body water were estimated as previously described^49^. Flies of the indicated genotype were maintained on normal or hypertonic media for 4 days. Adult flies from each genotype were anesthetized with CO_2_ and weighed. The abdomen of each fly was gently torn and hemolymph was blotted from the abdominal opening with a Kimwipe that had been slightly moistened with isotonic saline. Each fly was then weighed a second time, then dried for 1 h at 60°C and weighed a third time. Hemolymph volume was estimated by determining the reduction in mass following hemolymph blotting. Percentage of total body water and hemolymph were estimated.

### Desiccation resistance and starvation resistance

To evaluate desiccation resistance, 3-day-old male flies were placed in empty glass shell vials (10 flies per vial) and introduced into a Plexiglas desiccation chamber. The temperature was maintained at 24–25°C. The number of dead flies was scored at an hourly interval until all of the flies had died. For starvation resistance, 3-day-old male flies were introduced into vials containing 10 mL of 0.5% agar in groups of 10 flies per vial. The vials were changed to fresh medium every 48 h. Deaths were scored three times per day until all of the flies had died. Each genotype was tested three times^50^.

### Statistical analysis

Statistical significance was determined using an unpaired Student's *t*-test (two-tailed). *P*-values of less than 0.01 were considered significant.

## Author Contributions

Z.L. and H.L. designed the experiments; Z.L. performed the experiments; Z.L., H.L. and C.Q. wrote the manuscript. All authors reviewed the manuscript.
